# Editorial: Systems Biology Approach to the Immunology of Asthma and Allergy

**DOI:** 10.3389/fimmu.2022.857403

**Published:** 2022-02-14

**Authors:** Cagatay Karaaslan, Cristobalina Mayorga, Katja Baerenfaller, María M. Escribese

**Affiliations:** ^1^ Department of Biology, Molecular Biology Section, Hacettepe University, Ankara, Turkey; ^2^ Allergy Clinical Unit, Hospital Regional Universitario de Málaga-Instituto de Investigación Biomédica de Málaga (IBIMA), Red de Asma, Reacciones Adversas a Fármacos y Alergia (ARADyAL), Málaga, Spain; ^3^ Swiss Institute of Allergy and Asthma Research, Swiss Institute of Bioinformatics (SIAF), University of Zurich, Davos, Switzerland; ^4^ Institute of Applied Molecular Medicine (IMMA), Department of Basic Medical Sciences, Facultad de Medicina, Universidad San Pablo CEU, CEU Universities, Madrid, Spain

**Keywords:** allergy, system biology, asthma, immunology, biomarker

The prevalence of allergic diseases as well as their severity have risen progressively during the last decades, affecting a considerable percentage of the population worldwide. Asthma and allergies as a whole are heterogeneous and show a complex pattern of disease endotypes, a complicated balance between immune tolerance and allergic sensitization, and a progression of allergic diseases. The application of high-throughput technologies enables the profiling of genomes, epigenomes, transcriptomes, microbiomes and metabolomes to gain a better understanding of the immune mechanisms underlying asthma and allergies. The use of systems biology aims at combining and connecting the information on the different molecular components on the cell, tissue, or organ level to discover the properties of the full system. A system’s level understanding of the complex allergic diseases will then assist in implementing personalized medicine strategies for the treatment of allergic and asthmatic patients.

Allergic diseases are inflammatory diseases. A classical allergic reaction is characterized by the immune response of type 2 T helper cells (Th2) and group 2 innate lymphoid cells (ILC2s) that produce the cytokines IL-4, IL-5, and IL-13 that create an inflammatory environment affecting a variety of additional cell types (1, 2). Hyaluronan (HA) is a glycosaminoglycan present in epitelial tissue and a component of the exctracellular matrix, and high-molecular-weight (HMW-)HA is thought to have anti-inflammatory activity. To investigate whether a dysfunction of the *hyaluronan synthase 2* (*Has2*) gene can exacerbate asthma, Sherpa et al. used *Has2* heterozygous-deficient mice in a model of chronic ovalbumin sensitization and challenge. They found that *Has2* attenuation worsens eosinophilic airway inflammation, airway remodeling and steroid insensitivity, which highlights the importance of HAS2 and HMW-HA in controlling inflammation and airway remodelling.

A key mechanism that is associated with the onset of allergic diseases and the loss of peripheral immune tolerance is the dysregulation of regulatory T cells (Tregs) and an impaired balance between Treg and Th2 cells (3). Given the importance of Tregs in immune tolerance, it is important to have suitable experimental systems at hand to study their differentiation and immunological responses. In mice, CD4^+^ T cells expressing Foxp3 and RORγt are associated with tolerance against food antigens and regulatory functions against Th2 effector cells. Martinez-Blanco et al. investigated the effect of stimulating isolated murine CD4^+^CD25^-^ T cells with IL-2 and TGF-β, and found that this stimulation leads *in vitro* to the production of suppressive double-positive Foxp3^+^RORγt^+^ cells that excrete IL-17. Interestingly, the addition of retinoic acid (RA) led to the production of functionally repressive Foxp3^+^ cells and Treg cell lineage stabilization. As Foxp3^+^RORγt^+^ cells are found in the small intestinal and colonic lamina propria of mice, and gut microbiota stimulate the epithelial production of RA, this provides an interesting link to immune metabolism. Another immunomodulatory mediator that links metabolism to immune responses is the protein Adiponectin. Ramos-Ramírez et al. investigated the direct effect of Adiponectin on human Tregs and found that a subset of human Tregs expressed Adiponectin receptor 1 (AdipoR1). Treatment of CD4^+^ T cells with globular Adiponectin or the Adiponectin receptor antagonist induced the synthesis of anti-inflammatory cytokines such as IL-10 in AdipoR1+ Tregs, which was enhanced in an inflammatory type 2 cytokine environment. The anti-inflammatory properties of Adiponectin suggest that the adiponectin/AdipoR1 axis might be a novel therapeutic target.

One of the main aims of using systems biology approaches is the identification of biomarkers. To identify allergic rhinitis (AR) biomarkers, Yuan et al. characterized differences in the microbiome and serum metabolome of AR patients and healthy individuals in the upper respiratory tract. The microbiome analysis showed significant differences in the distribution of taxa in phylum and genus levels. Using predictive modelling, the the AR patients could be diagnosed based on 10 differential genera. In the serum metabolome, 26 differential metabolites and 16 disrupted metabolic pathways could be identified comparing AR patients and healthy individuals. The two most significantly altered metabolic pathways are linoleic acid and arachidonic acid metabolism. As described in the review by Rodriguez-Coira et al. on immune metabolism in allergic diseases, the arachidonic acid metabolites LTB4 and 5-HETE are potent lipid mediators. LTB4 gets released by macrophages and basophils upon binding of IgE to high-affinity Fc epsilon receptor I (FcϵRI), and increased production of LTB4 and 5-HETE was observed in airway macrophages from asthmatic patients. The review by Rodriguez-Coira et al. also summarises evidence on the pronounced effect of additional lipid mediators on allergy responses and on the key role of lipid metabolism in inflammation. Additionally, a summary over current approaches to characterize immunometabolism using omics and non-omics techniques is provided.

Anaphylaxis is one of the most frequent life-threatening systemic hypersensitivity reactions. Using a quantitative proteomics approach, Yuste-Montalvo et al. have characterized the effect of serum mediators released in an anaphylaxctic reaction on endothelial cells (ECs). To this end, cultivated microvascular ECs were treated with serum either from patients suffering from an anaphylactic reaction or from healthy non-allergic individuals, followed by protein labelling and mass spectrometry measurements. Differential quantitative protein analysis revealed a subset of 47 highly altered proteins, and that proteins of thee coagulation and complement systems are increased in ECs treated with serum from anaphylactic patients. Following further analyses, the complement system was identified as the main altered category in EC-anaphylaxis, pointing to a of the endothelial niche in the activation of the complement system during anaphylaxis.

Respiratory allergic diseases are characterized by the the clinical history of airway allergic symptoms, the positive response to nasal or bronchial allergen challenge, and positive skin prick tests or the presence of serum allergen-specific IgE. Testera-Montes et al. review the current evidence on local allergic rhinitis (LAR), which in contrast to the allergy-based classification of airway diseases focuses on the presence of airway allergen-specific reactivity and is characterized by negative atopy test results. LAR show a distinct phenotype that does not evolve to systemic atopy over time, but progresses towards aggravation of symptoms and comorbidities such as conjunctivitis and asthma. Yet the allergen-specific reactivity in LAR is still associated with allergic inflammation, and the effect of allergen-specific immunotherapy seems to be similar as in atopic respiratory allergy. Testera-Montes et al. thereofore postulate that atopy and allergy represent two different phenomena that require distinct identification methods.

The major aims of Systems Biology approaches in the immunology of asthma and allergy field are the molecular characterization of the immune responses, the identification of potential biomarkers and therapeutic targets, and the detection of disease endotypes and patient theratypes. The articles collected in this Research Topic span this broad with of applications and thereby provides interesting insights into the capacities of the Systems Biology approach to provide insights into a biomedically relevant field.

## Author Contributions

All the authors have contributed in the design, writing, and discussion of this editorial.

## Funding

This work was supported by ISCIII (PI18/01467), cofounded by FEDER “Investing in your future” for the thematic network and co-operative research centres ARADyAL RD16/0006/0015; as well as by the grant from Ministerio de Ciencia, Innovación y Universidades co-financed with FEDER RTI2018-095166-B-I00.

## Conflict of Interest

The authors declare that the research was conducted in the absence of any commercial or financial relationships that could be construed as a potential conflict of interest.

## Publisher’s Note

All claims expressed in this article are solely those of the authors and do not necessarily represent those of their affiliated organizations, or those of the publisher, the editors and the reviewers. Any product that may be evaluated in this article, or claim that may be made by its manufacturer, is not guaranteed or endorsed by the publisher.
